# Distribution of Peripheral Lymphocyte Populations in Primary Sjögren's Syndrome Patients

**DOI:** 10.1155/2015/854706

**Published:** 2015-05-18

**Authors:** Gintaras Sudzius, Diana Mieliauskaite, Almantas Siaurys, Rita Viliene, Irena Butrimiene, Dainius Characiejus, Irena Dumalakiene

**Affiliations:** ^1^Department of Immunology, State Research Institute Centre for Innovative Medicine, LT-08409 Vilnius, Lithuania; ^2^Department of Innovative Diagnostic Treatment and Health Monitoring Technology, State Research Institute Centre for Innovative Medicine, LT-01102 Vilnius, Lithuania; ^3^Faculty of Medicine, Vilnius University, LT-03101 Vilnius, Lithuania; ^4^Department of Pathology, Forensic Medicine and Pharmacology, Faculty of Medicine, Vilnius University, LT-03101 Vilnius, Lithuania; ^5^Department of Chemistry and Bioengineering, Faculty of Fundamental Sciences, Vilnius Gediminas Technical University, LT-10223 Vilnius, Lithuania

## Abstract

Purpose of this study was to evaluate the lymphocyte populations' distribution changes in peripheral blood of patients with primary Sjögren's syndrome (pSS). Lymphocyte populations' distribution changes in peripheral blood of pSS patients were investigated in 52 patients with pSS and in 28 healthy controls by flow cytometry. We found decreased absolute count of CD3^+^ T cell population in pSS patients. Analysis of CD4^+^ T cell population showed significant proportion and absolute count differences in pSS patient's blood with SSA/SSB antibodies (Abs) in comparison to controls. No significant differences were observed analyzing CD4^+^ and CD8^+^ Treg subpopulation. Proportion and absolute counts of Th17 cells were significantly lower in pSS patient's blood. Absolute counts of CD8^+^ T cells were significantly lower in pSS patients in comparison to controls and also impaired proportion and absolute counts of CD8^+^ subpopulations according to CD27^+^ and CD57^+^ were observed. Absolute counts of NKT and NK cells were decreased in pSS with Abs. B cells proportion was increased only in blood of pSS with Abs. Lymphocyte distribution impairment can be due to genetically determined lymphopenia or lymphocyte migration from periphery to inflammatory sites or/and increased susceptibility to apoptosis.

## 1. Introduction

Primary Sjögren's syndrome (pSS) is a systemic autoimmune disorder that affects secretory organs and is characterized by ocular and mouth dryness, fatigue, and pain, as well as extra-glandular manifestations that reveal the severity of this disorder [1, 2]. Patients with pSS also present broad spectrum analytical features (cytopenias, hypergammaglobulinemia, and cryoglobulins). Biological signatures of the disease are B-lymphocyte activation, which could be triggered by the dysregulation of B-cell activating factor (BAFF) [1]. It is suggested that BAFF is influential in driving antibody production in autoimmune diseases [2]. One of the objective classification criteria for pSS is serum SSA/SSB antibodies (Abs) [2]. Recent research studies suggest that these antibodies may also be the biomarkers of disease activity [3]. Some studies indicate that anti-SSA/SSB seropositive patients have the increased amount of B-cell activation markers, such as BAFF, free immunoglobulin light chain, beta-2 microglobulin, and IgG [3–7]. Thus, the spectrum of the disease ranges widely from minimal local symptoms of the eyes and oral mucosa to systemic involvement and development of malignant lymphoma; the latter are being the most worrisome complication of pSS [2]. Pathophysiology of Sjögren's syndrome is not yet fully understood. Recently, much attention has been focused on the relationship between innate responses and subsequent activation of specific adaptive-immunity in an attempt to understand subsequent immune dysregulation [8–10]. Specific cytotoxic lymphocyte populations can lead to the formation of autoimmune diseases, whereas suppressive/regulatory cell populations may lead to suppression of autoimmunity and disease remission [11, 12]. However, the pathological role of T cells in pSS remains to be elucidated.

The aim of the study was to perform a detailed quantitative analysis of peripheral blood CD4^+^ and CD8^+^ T lymphocyte subpopulations in patients with Sjögren's syndrome with special emphasis on Treg, Th17, NKT lymphocytes, NK cells, and B cells and expression of CD57 and CD27 markers on CD8^high^ lymphocytes.

## 2. Patients and Methods

### 2.1. Patients

In total, 52 patients with pSS and 28 healthy controls were recruited at the State Research Institute Center for Innovative Medicine for this study. Patients with pSS were grouped in two groups: pSS Abs^−^ group, 29 without anti-SSA and/or anti-SSB Abs, and pSS Abs^+^ group, 23 patients with anti-SSA and/or anti-SSB Abs. The average age of the patients groups and healthy controls was accordingly: 57 ± 13 years, 56 ± 13 years, and 53 ± 11 years. The majority of enrolled patients in our study were Lithuanian women. Only 1 Lithuanian man (1 in pSS Abs^+^ group) was enrolled in pSS group. Nobody of the control group had connective tissue diseases, anti-SSA or anti-SSB Abs. Primary SS was diagnosed according to American-European Consensus Group Classification criteria for Sjögren's syndrome [2]. All patients underwent serologic evaluations, which included test for the presence of antibodies against SSA and SSB, Schirmer's *I* test, unstimulated whole salivary flow test, and histology of minor salivary glands. Disease activity was assessed using EULAR Sjögren's syndrome disease activity index (ESSDAI) [13] and EULAR Sjögren's syndrome patient reported index (ESSPRI) [14]. The characteristics of the pSS patients included in the study are summarized in Table 1. Informed and written consent was obtained from all participants of this study. The study has been approved by the Lithuanian Bioethics Committee (no. 158200-03-299-73).

### 2.2. Lymphocyte Populations' Proportion and Absolute Counts Determination in Peripheral Blood

Blood samples were collected from heparinized venous blood. Absolute counts of white blood cells (WBC) were determined with a haemocytometer and used for calculation of absolute numbers of lymphocyte populations (numbers of cells/*μ*L peripheral blood). For cell surface staining, the following mAbs were used: anti-CD3 FITC (Exbio, Czech); anti-CD4 PerCP (BD, USA); anti-CD8-PerCP (BD); anti-CD-16+56-PE (Exbio, Czech); anti-CD-19 PerCP (Exbio, Czech); anti-CD57 FITC (BD, USA); and anti-CD27-APC (Exbio, Czech). For isotype controls staining, mouse anti-IgG1-FITC (BD, USA), IgG1-PerCP (BD, USA), and IgG2a-PE (BD, USA) were used. Staining was performed at room temperature for 30 min. Cell staining was followed by red blood cell lysis using Pharm Lyse (BD, USA) lysing solution for 15 min at room temperature in the dark. Leukocytes were then centrifuged (500 g for 10 min) and washed two times with CellWash (BD, USA) and resuspended in FBS (BD, USA). Samples were examined immediately after staining without fixation.

For the evaluation of intracellular cytokine IL-17A of CD4^+^ T cells, 1 mL of whole heparinized blood was diluted 1 : 2 in RPMI-1640 supplemented with 80 mg/L gentamycin and 2 nM glutamine. Cells were stimulated using 50 ng/mL phorbol-myristate-acetate (Sigma Aldrich, St. Louis, MO, USA) and 1 ng/mL ionomycin (Sigma Aldrich) in the presence of 0.7 *μ*L/mL monensin (GolgiStop (BD, USA)) for 4.5 h at 37°C in an atmosphere containing 5% CO_2_. Unstimulated cells served as controls. Following stimulation, cells were stained for CD4 for 30 min at room temperature. Cell staining was followed by red blood cell lysis using Pharm Lyse (BD, USA) lysing solution for 15 min at room temperature in the dark. Leucocytes were then centrifuged (at 500 g for 10 min) and washed two times with CellWash (BD). Then, cells were fixed and permeabilized with Cytofix/Cytoperm (BD, USA) solution for 20 min, washed two times with Perm/Wash (BD, USA) solution, and incubated further for 30 min in the dark with the specific mAbs anti-IL17A APC (BD, USA) and isotypic control anti-IgG1 APC (BD, USA). Following the incubation with mAbs, the cells were washed two times with Perm/Wash solution and resuspended in FBS (BD, USA).

For the evaluation of intracellular FoxP3 marker of CD4^+^ and CD8^+^ T cells, cells were stained with anti-CD4 PerCP (BD, USA), anti-CD25 FITC (Exbio, Czech), and anti-CD8-PerCP (BD, USA) for 30 min at room temperature. Cell staining was followed by red blood cell lysis using Pharm Lyse (BD, USA) solution for 15 min at room temperature in the dark. Leucocytes were then centrifuged (at 500 g for 10 min) and washed two times with CellWash (BD, USA). Then, cells were fixed and permeabilized with Cytofix/Cytoperm (BD, USA) solution for 20 min, washed two times with Perm/Wash (BD, USA) solution, and incubated further for 30 min in the dark with the specific mAbs anti-FoxP3-PE (BD, USA) and isotypic controls anti-IgG1-FITC (BD, USA), IgG1-PerCP (BD, USA), and IgG2a-PE (BD, USA). Following the incubation with mAbs, the cells were washed two times with Perm/Wash (BD, USA) solution and resuspended in FBS (BD, USA). Lymphocytes were gated and separated based on their morphological properties.

Flow cytometry was performed on FACSCalibur flow cytometer (BD Biosciences, San Jose, CA, USA) calibrated with CaliBRITE beads (BD Biosciences, San Jose, CA, USA) using CELL-Quest software (BD Biosciences, San Jose, CA, USA). Data for each sample were acquired until 100,000 leukocytes were analyzed.

### 2.3. Determination of BAFF Level in Serum

Serum samples were analyzed using commercial BAFF, Soluble (human) ELISA Kit (hypersensitive) (AdipoGen, Switzerland). From collected blood samples, serum was separated and stored at −80°C until analysis. Serum dilutions and enzyme-linked immunoassay was carried out in the strict accordance with the manufacturer's instructions and sets recommendations. The results were evaluated by spectrophotometer (BioTek Instruments, USA). The concentrations of analytes in ELISA assays were quantified using standard curves. A regression analysis was performed to derive an equation that was then used to predict the concentration of the unknown samples with Gen5 Microplate Data Collection & Analysis Software (BioTek Instruments, USA). The results are shown in Table 1.

### 2.4. Statistics

Statistical differences were analyzed using the Mann-Whitney *U* test. Correlations were assessed by the Spearman's rank test using standard program GraphPad Prism 5.0 software (GraphPad Software, San Diego, CA, USA). *P* values less than 0.05 were considered significant.

## 3. Results

### 3.1. Main Lymphocyte Populations

**Figure 1:**
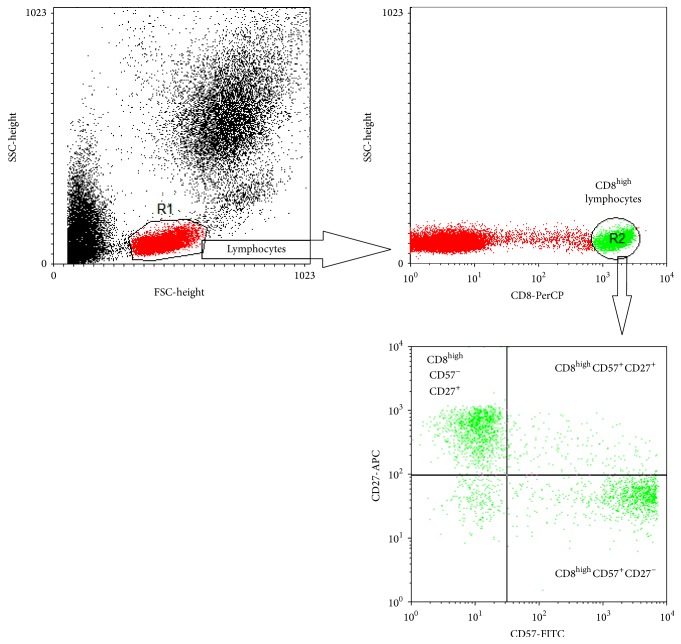
Representative dot plots. CD8^high^ lymphocytes in the CD8^+^ subset were determined in the flow cytometric SSC/CD8-PerCP dot plot and percentages of CD8^high^CD57^−^CD27^+^ lymphocytes were determined in gated CD8^high^ subset in the flow cytometric CD57-FITC/CD27-APC dot plot.

We found that absolute count of CD3^+^ T cell population was significantly decreased in pSS patients in comparison to healthy controls. No differences were observed between pSS groups. Significant decrease of CD3^+^ cells was found in pSS Abs^−^ (*P* = 0.027) and pSS Abs^+^ (*P* = 0.0002) groups when compared to controls; however, no differences in the CD3^+^ cells proportion of WBC were found between pSS groups. Analysis of CD4^+^ T cell population showed significant proportion differences in pSS Abs^+^ patient's blood when compared to pSS Abs^−^ (*P* = 0.036) and control group (*P* = 0.0036). But absolute counts of CD4^+^ cells were significantly lower in both pSS groups than in control group, pSS Abs^−^ (*P* = 0.015) and pSS Abs^+^ (*P* < 0.0001), and also absolute counts of CD4^+^ cells were significantly lower in pSS Abs^+^ than in pSS Abs^−^ (*P* = 0.01) patients' blood. No significant differences in the proportion of CD8^+^ T cell population were found between pSS patients and controls. But analysis of absolute counts showed significantly lower counts of CD8^+^ T cells in pSS Abs^−^ (*P* = 0.014) and pSS Abs^+^ (*P* = 0.006) in comparison to controls; no differences were found between pSS groups (Table 2, Figure 1). Analysis of NKT (CD3^+^CD16/56^+^) and NK (CD3^−^CD16/56^+^) cells showed only decreased absolute counts in pSS Abs^+^ group (resp., *P* = 0.009 and *P* = 0.036) in comparison to controls. Increased proportion of CD3^−^CD19^+^ (B cells) in pSS Abs^+^ (*P* = 0.045) was found when compared to controls, but no differences were found analyzing absolute counts among pSS groups and controls (Table 2).

### 3.2. CD4^+^ Lymphocyte Subpopulations

**Figure 2:**
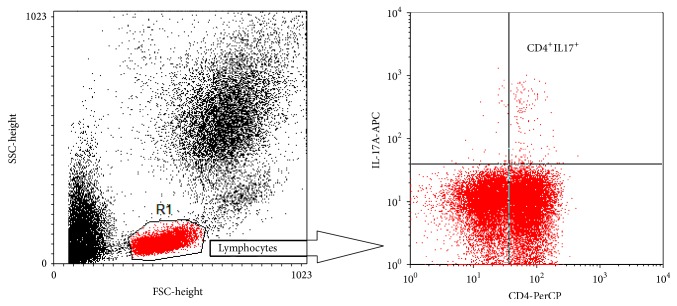
Representative dot plots. CD4^+^IL-17A^+^ positive cells were determined on CD4-PerCP versus IL-17A-APC dot plot by gating on lymphocyte on the forward-scatter versus side-scatter dot plot.

Analysis of CD4^+^ lymphocyte subpopulations according to their expression of CD25 and FoxP3 markers showed significant reduced absolute counts of CD4^+^CD25^+^ (*P* = 0.036) and CD4^+^ CD25^low^FoxP3 (*P* = 0.017) cells when comparing pSS Abs^+^ with controls. No significant differences were observed analyzing other CD4^+^ subpopulations according to their expression of CD25 and FoxP3 markers (Table 3). Analysis of CD4^+^IL-17A^+^ (Th17) cells showed significant lower proportion and absolute counts of these cells in pSS patients in comparison to control group's results (Figure 2). Proportion was significantly altered in pSS Abs^−^ (*P* = 0.0003) and pSS Abs^+^ (*P* = 0.004), and also absolute counts of Th17 cell were significantly lower in pSS Abs^−^ (*P* < 0.0001) and pSS Abs^+^ (*P* < 0.0001) patients' blood in comparison to controls. No significant differences were found analyzing proportion and absolute counts of this subpopulation between pSS groups (Table 3, Figure 1).

### 3.3. CD8^+^ Lymphocyte Subpopulations

CD8^high^ lymphocyte population was differentiated to subpopulations according to the markers CD57 and CD27 that defines replicative senescence (Figure 1). Analysis of CD8^high^CD57^+^CD27^−^ subpopulation showed no differences in proportion and absolute counts among pSS and control groups. In pSS patients with Abs, we observed significantly increased proportion (*P* = 0.032) and absolute counts (*P* = 0.011) of CD8^high^CD57^+^CD27^+^ subpopulation in comparison to pSS without Abs. No significant differences were found when comparing results of pSS Abs^+^ and pSS Abs^−^ to controls. CD8^high^CD57^−^CD27^+^ population's proportion was significantly reduced only in pSS without Abs patients' blood (*P* = 0.026) when compared to controls and no significant differences between pSS groups were observed. Absolute counts of this subpopulation were significantly reduced both pSS Abs^−^ (*P* = 0.0003) and pSS Abs^+^ (*P* = 0.005) in comparison to controls. Proportion of CD8^high^ subpopulation lacking CD57 and CD27 markers (CD8^high^CD57^−^CD27^−^) was significantly increased only in pSS Abs^−^ patients' blood when compared to controls. Differences in proportion or absolute counts of subpopulation expressing FoxP3 marker were not observed (Table 4).

### 3.4. Correlation between Cell Populations' Changes and Clinical Parameters in pSS Abs^−^ Patients

Focus score correlated with absolute counts of CD3^+^ (*P* = 0.047, *r* = 0.474), CD4^+^ (*P* = 0.033, *r* = 0.503), B cell (*P* = 0.023, *r* = 0.532), and CD8^+^CD57^−^CD27^+^ (*P* = 0.016, *r* = 0.560) cell populations. ESSPRI correlated with NK (*P* = 0.034, *r* = 0.501). ESSDAI correlated with NK (*P* = 0.043, *r* = 0.482) and CD4^+^CD25^high^FoxP3 (*P* = 0.047, *r* = 0.474) cell absolute counts. In pSS patients without Abs, we found that Schirmer's *I* test correlated with proportion (*P* = 0.033, *r* = 0.504) and absolute counts (*P* = 0.015, *r* = 0.562) of Th17 cells. Serum BAFF concentration correlated with proportion of CD8^+^ (*P* = 0.032, *r* = 0.415), NK (*P* = 0.009, *r* = 0.491), and CD8^high^CD57^+^CD27^−^ (*P* = 0.014, *r* = 0.469) cell populations and negatively correlated with B cells (*P* = 0.026, *r* = −0.429), CD8^high^CD57^+^CD27^+^ (*P* = 0.036, *r* = −0.404), and CD8^high^CD57^−^CD27^+^ (*P* = 0.009, *r* = −0.493). Analyzing absolute counts of cells populations observed negative correlation between BAFF and CD4^+^ (*P* = 0.025, *r* = −0.430), B cell (*P* = 0.007, *r* = −0.509), CD8^high^CD57^+^CD27^+^ (*P* = 0.028, *r* = −0.424), and CD8^high^CD57^−^CD27^+^ (*P* = 0.017, *r* = −0.454) cells.

### 3.5. Correlation between Cell Populations' Changes and Clinical Parameters in pSS Abs^+^ Patients

Schirmer's *I* test correlated with proportion of B cells (*P* = 0.038, *r* = 0.467) and CD8^high^CD57^−^CD27^+^ (*P* = 0.006, *r* = 0.589) and negatively correlated with CD3^+^ (*P* = 0.006, *r* = −0.590) and CD8^high^CD57^−^CD27^−^ (*P* = 0.033, *r* = −0.479) cells. Also correlation between Schirmer's *I* test and absolute count of CD8^high^CD57^−^CD27^+^ (*P* = 0.021, *r* = 0.512) and negative correlation with CD8^high^CD57^−^CD27^−^ (*P* = 0.017, *r* = −0.525) cell counts were observed. Unstimulated salivary flow rate correlated with proportion of CD8^+^ (*P* = 0.017, *r* = 0.525) and Th17 (*P* = 0.015, *r* = 0.535) and also negatively correlated with absolute counts of CD3^+^ (*P* = 0.011, *r* = −0.555) and CD4^+^ (*P* = 0.017, *r* = −0.526) cells. Focus score negatively correlated with proportion and absolute counts of CD8^high^CD57^+^CD27^−^ cells, respectively, (*P* = 0.020, *r* = −0.517) and (*P* = 0.040, *r* = −0.462). ESSPRI correlated with proportion of CD3^+^ (*P* = 0.015, *r* = 0.535), CD4^+^FoxP3 (*P* = 0.044, *r* = 0.454), and CD4^+^CD25^+^FoxP3 (*P* = 0.041, *r* = 0.461) cells and negatively correlated with proportion of CD4^+^CD25^+^ (*P* = 0.026, *r* = −0.496) and CD4^+^CD25^high^ (*P* = 0.043, *r* = −0.456) cells. ESSDAI negatively correlated with proportion of Th17 (*P* = 0.001, *r* = −0.675) cells and also with absolute counts of CD8^+^ (*P* = 0.042, *r* = −0.459), Th17 (*P* = 0.001, *r* = −0.673), and CD8^high^CD57^+^CD27^+^ (*P* = 0.028, *r* = −0.492) cells. No correlation was observed between serum BAFF concentration and cell populations changes in pSS patients with Abs.

## 4. Discussion

Despite systemic B-cell hyperactivity, T and B cells constitute the vast majority of infiltrating mononuclear cells at the minor salivary glands inflammatory lesions of pSS, with their prevalence varying according to the severity of the infiltrates. The majority of these T cells are CD4^+^ and show an activated phenotype. CD8^+^ T cells with cytotoxic activity, as manifested by their expression of granzymes, constitute around 15% of infiltrating cells. T cells predominate in mild lesions, whereas in severe lesions B cells constitute the main population. The prevalence of CD4^+^ T cells decreases with lesion severity, whereas the prevalence of CD8^+^ T cells remains unchanged. The prevalence of regulatory T cells associates with lesion severity, with the higher values to be observed at intermediate lesions. NK cells comprise a small but considerable portion of the infiltrating mononuclear cells, and their percentage correlates with the grade of the lesions [15, 16].

In our study, T lymphocyte identification by CD4 and CD8 markers showed a statistically significant decrease in the absolute counts of CD4^+^ and CD8^+^ T lymphocytes in the peripheral blood of pSS patients in comparison to the control group. This shows that the decline of CD3^+^ T lymphocyte population in the peripheral blood of pSS patients is influenced by a decrease of both CD4^+^ and CD8^+^ T lymphocyte absolute counts. The decrease of the total amount of CD4^+^ T lymphocytes in the peripheral blood of pSS patients is also confirmed by other authors [17]. In some pSS patients, low counts of CD4^+^ lymphocytes or their dysfunction in peripheral blood maybe due to anti-CD4 antibodies. These autoantibodies in some pSS patients' serum were identified by Henriksson and colleagues [18]. The fact is that the proportion of CD4^+^ was lower only in our pSS patients with Abs, and no differences in proportion of CD3^+^ and CD8^+^ in all pSS patients were observed; let us think that this lymphopenia can also be genetically determined. Apoptosis may also play a role in the pathogenesis of some extraglandular manifestations of pSS and peripheral CD4^+^ lymphocytopenia [19, 20].

Th17 cells also appear to play a role in the development of pSS. Studies in patients with pSS and animal models of pSS have identified the presence of IL-17 in the lymphocytic infiltrates of the exocrine glands, as well as higher levels of circulating IL-17 in both serum and saliva [8, 21, 22]. On the one hand, our finding that Th17 lymphocyte counts decreased in the peripheral blood of patients with Sjögren's syndrome is quite unexpected. On the other hand, this result may be explained by the redistribution of Th17 lymphocytes, that is, increasing their concentration in tissues (salivary glands) and decreasing concentration in peripheral blood [8, 10]. Also, we cannot dismiss presumable apoptosis of peripheral Th17 cells.

Treg lymphocytes are characterized by autoimmune reaction-inhibiting properties [15, 23]. So they should be reduced in patients with SS [24]. However, in this study, we have not find more distinct Treg cells changes in the peripheral blood of pSS patients. No statistical significant differences were found analyzing CD4^+^CD25^high^FoxP3 cells, CD4^+^CD25^high^, or CD4^+^CD25^+^FoxP3 cells. Alike results were published by Sarigul and colleagues [25]. Sometimes it is hard to define what is what, when conflicting results have been reported. One of the problems is that different authors as Treg population define different pools of CD4^+^ cells. Some researchers uses two markers CD4^+^ and CD25^high^ to identify Treg cells [26], while others also uses FoxP3 marker [15]. This is why we have checked more pools of CD4^+^ that some authors define as Treg cells.

The role of cytotoxic T cells in pSS pathogenesis has not been studied in detail. Autoreactive cytotoxic T cells are seen in pSS targeting autoantigens. CD8^+^ T-cell deficiency is a feature of many chronic autoimmune diseases, including multiple sclerosis, rheumatoid arthritis, systemic lupus erythematosus, Sjögren's syndrome, systemic sclerosis, ulcerative colitis, Crohn's disease, psoriasis, etc. It also occurs in blood of healthy relatives of patients with autoimmune diseases, suggesting that it is genetically determined. These cells play critical roles in purging acute infections, limiting persistent infections, and conferring life-long protective immunity. CD8^+^ T cell deficiency can prompt the development of chronic autoimmune diseases by impairing CD8^+^ T cell control of virus infection [27]. It is known that viral infections are the best candidates for the role of environmental triggers to autoimmune reactions [28]. It is proposed that, after activation in peripheral lymphoid organs by cross-reacting foreign antigens, autoreactive T cells enter the target organ where they are reactivated by B cells which provide costimulatory survival signals, thereby inhibiting the activation-induced T-cell apoptosis which normally occurs when autoreactive T cells enter the target organ [27]. Understanding CD8^+^ T memory effector cells differentiation is essential for studying how virus-specific CD8^+^ T cells control viral infection. Distinct stages of virus-specific CD8^+^ T memory effector cells differentiation have been extensively characterized by phenotypic and functional analyses. Primed virus-specific CD8^+^ T cells typically differentiate from the least mature memory stage (CD27^+^) to the most mature effector stage when they start to lose CD27 and obtain CD57 marker and eventually become terminally differentiated effector cells which can be further defined by CD57 expression [29]. According to some studies, last stage of CD8^+^ cells differentiation seems to be CD8^+^CD27^−^CD57^−^ T cells subset with high perforin and killing activity. Is this the true end-stage or terminally-differentiated state of cytotoxic T cells? This fact is still unclear [30]. We observed increased proportion of this population in pSS patients, but significant differences were observed only in pSS without Abs group. In pSS with Abs group, negative correlation between CD8^high^CD27^−^CD57^−^ T cells subset absolute count and Schirmer's *I* test results was observed. This connection proposes that this subset can be involved in a pathogenic process which appears in the glandular tissue. Impaired proportion and absolute counts of CD8^high^CD27^+^CD57^−^ T cells in blood of pSS patients can be the reason of lower counts of CD8^+^ in blood of pSS patients. It is not known whether the proportion and absolute counts of this population are downregulated in blood by migration to inflammatory sites, or this can be due to increased apoptosis of these cells. There is hypothesis that more mature effector stage (CD8^high^CD27^+^CD57^+^ and CD8^high^CD27^−^CD57^+^) cells with lower capacity of proliferation are more resistant to apoptosis than least mature memory stage CD8^high^CD27^+^CD57^−^ T cells [29–31]. All these observed changes in CD8^+^ T cell subpopulations rearrangement prove that these subpopulations actively participate in pathological processes of pSS.

NKT lymphocytes and NK cells might function as regulatory T cells and are one of the autoimmune process preventing chains [32, 33]. According to literature, in patients with autoimmune rheumatic diseases, the decreased NKT and NK cell counts and functional characteristics are associated with the progression of autoimmune process and autoantibody production [34–37]. Nevertheless Szodoray et al. published results where they identified higher proportion of these cells in blood of pSS patients than in control group [38]. Our investigation of these populations showed a significant decrease of NKT and NK-cell absolute counts in the peripheral blood of pSS patients with Abs; however, the fact that a proportion of these cells were similar with the controls can indicate that lower absolute counts can be due to genetically determined lymphopenia. There is also possibility that low counts of these cell populations in periphery can be by reason of overall lymphocyte population migration to inflammatory sites or/and apoptosis.

In conjunction with the classical CD4^+^ Tregs, we were also investigating CD8^+^ suppressor cells that express FoxP3 marker, as FoxP3 confers suppressive properties and is confined to regulatory T cells. CD8^+^FoxP3 cells represent a new regulatory population and ability of these CD8^+^FoxP3 Treg to suppress CD8^+^ responses far more effectively than CD4^+^FoxP3 Treg [39]. This was shown in mice after experimental allogeneic bone marrow transplantation. Our study results did not show any significant differences in proportion or absolute count changes on these cells in pSS patients' peripheral blood in comparison to healthy controls.

We found negative correlation between BAFF and T and B cells in pSS patients without Abs. Increase of BAFF in serum can be due to negative regulation of BAFF secretion by monocytes [40]. This fact can indicate tight control of BAFF secretion. Whereas we do not found correlation between BAFF and lymphocyte populations changes in pSS patients with Abs group, what can be the indication of uncontrolled BAFF secretion and its homeostasis disturbance?

Despite recent knowledge, in many respects, the role of T cells and their subsets in pSS remains unexplained. Are cells in the infiltrate specific, or maybe many of them are just bystanders (with nonactivated phenotype) recruited from the periphery to the inflammatory sites? T cells undergo expansion within the gland, or does this occur elsewhere with subsequent migration? Is there migration in and out of the gland, or do T cells remain in the infiltrates once they arrive? Pointers to these questions could help us understand which processes are going on periphery. All this together could help us to understand pathogenesis of the primary SS.

One of future projects should be the immunohistochemistry for assessing cell populations' changes in salivary glands in parallel with blood analysis and apoptosis markers. Such analysis could help better to define changes of cell populations in periphery, is this due migration to the inflammatory sites or increased apoptosis, or maybe both.

## Figures and Tables

**Table 1:** Clinical and serological characteristics of pSS patients.

Features	^1^pSS Abs^−^	^2^pSS Abs^+^	*P* ^*^
	(*n* = 29)	(*n* = 23)	I-II
Age, mean ± SD years	56 ± 12	56 ± 13	0.873
Schirmer's *I* test (mm/5 min), mean ± SD	3.17 ± 1.20	1.25 ± 1.12	<0.0001
Unstimulated salivary flow (mL/15 min), mean ± SD	1.22 ± 0.20	0.83 ± 0.39	0.002
Biopsy focus score	1.17 (1/2)	2.40 (1/3)	<0.0001
(number of lymphocytic foci/4 mm^2^), mean (min/max)			
Anti-SSA^+^, *n* (%)	0 (0)	6 (26)	
Anti-SSB^+^, *n* (%)	0 (0)	2 (9)	
BAFF, mean ± SD, ng/mL	4230 (1748)	5014 (2518)	0.187
Anti-SSA/SSB^+^, *n* (%)	0 (0)	15 (65)	
ESSPRI, mean (min/max)	8.39 (6/10)	9.1 (7/10)	0.075
ESSDAI, mean (min/max)	24.83 (21/30)	33.50 (27/39)	<0.0001

^*^Mann-Whitney test. ^1^pSS Abs^−^: pSS patients without anti-SSA/SSB; ^2^pSS Abs^+^: pSS patients with anti-SSA and/or anti-SSB. ESSDAI: EULAR Sjögren's syndrome disease activity index; ESSPRI: EULAR Sjögren's syndrome patient reported index.

**Table 2:** Distribution of lymphocyte populations in peripheral blood of pSS patients and controls.

Patients	^1^I-controls	^2^II-pSS Abs^−^	^3^III-pSS Abs^+^	*P* ^*^	*P* ^*^	*P* ^*^
				I-II	I-III	II-III
Main lymphocyte populations						
CD3^+^ T cells						
%^4^	75.36 ± 6.93	72.16 ± 9.81	73.03 ± 8.47	NS	NS	NS
A. count^#^	2530 ± 811.0	2154 ± 1133	1868 ± 1052	**0.027**	**0.0002**	NS
CD4^+^ T cells						
%^4^	49.33 ± 9.12	46.20 ± 8.49	39.37 ± 12.39	NS	**0.0036**	**0.036**
A. count^#^	1688 ± 654.9	1391 ± 804.3	1089 ± 975.4	**0.015**	**<0.0001**	**0.01**
CD8^+^ T cells						
%^4^	26.73 ± 7.29	26.25 ± 8.82	29.87 ± 11.64	NS	NS	NS
A. count^#^	939 ± 326.4	760.5 ± 433.5	690.2 ± 259.6	**0.014**	**0.006**	NS
B cells						
%^4^	11.86 ± 4.43	13.82 ± 9.13	14.52 ± 5.15	NS	**0.045**	NS
A. count^#^	401.3 ± 216.5	351.2 ± 197.8	393.5 ± 294.1	NS	NS	NS
NK						
%^4^	12.19 ± 5.67	12.99 ± 6.84	12.10 ± 6.05	NS	NS	NS
A. count^#^	415.4 ± 186.7	365.7 ± 210.7	306.1 ± 201.7	NS	**0.036**	NS
NKT						
%^4^	6.79 ± 4.26	8.44 ± 8.51	6.36 ± 5.86	NS	NS	NS
A. count^#^	223.4 ± 132.0	206.7 ± 168.6	137.7 ± 127.1	NS	**0.009**	NS

^1^I group: healthy controls; ^2^II group: pSS patients without anti-SSA/SSB; ^3^III group: pSS patients with anti-SSA and/or anti-SSB; %^4^: proportion of all CD4^+^ lymphocytes; A. count^#^: absolute count of cells in 1 *μ*L of blood. ^*^Mann-Whitney test. NS: not significant.

**Table 3:** Distribution of CD4^+^ lymphocyte subpopulations in peripheral blood of pSS patients and controls.

Patients	^1^I-controls	^2^II-pSS Abs^−^	^3^III-pSS Abs^+^	*P* ^*^	*P* ^*^	*P* ^*^
				I-II	I-III	II-III
CD4^+^ lymphocyte populations						
CD4^+^CD25^+^						
%^4^	10.00 ± 10.94	9.72 ± 6.1	11.64 ± 9.38	NS	NS	NS
A. count^#^	183.6 ± 222.3	132.7 ± 106.0	106.1 ± 75.89	NS	**0.036**	NS
CD4^+^CD25^high^						
%^4^	1.97 ± 1.342	2.45 ± 1.71	2.71 ± 1.83	NS	NS	NS
A. count^#^	32.13 ± 25.88	31.81 ± 25.92	24.39 ± 13.50	NS	NS	NS
CD4^+^FoxP3						
%^4^	4.56 ± 4.73	5.19 ± 6.69	5.012 ± 4.68	NS	NS	NS
A. count^#^	75.25 ± 82.62	59.45 ± 72.83	62.62 ± 105.0	NS	NS	NS
CD4^+^CD25^+^FoxP3						
%^4^	1.12 ± 2.09	1.10 ± 0.90	0.90 ± 0.63	NS	NS	NS
A. count^#^	19.12 ± 42.33	13.19 ± 10.79	10.21 ± 13.65	NS	NS	NS
CD4^+^CD25^high^FoxP3						
%^4^	0.27 ± 0.34	0.36 ± 0.31	0.35 ± 0.27	NS	NS	NS
A. count^#^	4.45 ± 6.9	4.039 ± 2.91	3.26 ± 2.81	NS	NS	NS
CD4^+^CD25^low^FoxP3						
%^4^	0.84 ± 1.75	0.78 ± 0.73	0.57 ± 0.51	NS	NS	NS
A. count^#^	14.57 ± 35.46	9.64 ± 9.612	6.71 ± 12.78	NS	**0.017**	NS
Th17						
%^4^	2.08 ± 1.31	0.85 ± 1.08	1.51 ± 2.89	**0.0003**	**0.004**	NS
A. count^#^	34.44 ± 21.02	9.12 ± 10.89	10.89 ± 22.71	**<0.0001**	**<0.0001**	NS

^1^I group: healthy controls; ^2^II group: pSS patients without anti-SSA/SSB; ^3^III group: pSS patients with anti-SSA and/or anti-SSB; %^4^: proportion of all CD4^+^ lymphocytes; A. count^#^: absolute count of cells in 1 *μ*L of blood. ^*^Mann-Whitney test. NS: not significant.

**Table 4:** Distribution of CD8^+^ lymphocyte subpopulations in peripheral blood of pSS patients and controls.

Patients	^1^I-controls	^2^II-pSS Abs^−^	^3^III-pSS Abs^+^	*P* ^*^	*P* ^*^	*P* ^*^
				I-II	I-III	II-III
CD8^+^ lymphocyte population						
CD8^high^CD57^+^CD27^+^						
%^4^	5.306 ± 3.899	5.329 ± 5.431	8.239 ± 8.239	NS	NS	**0.032**
A. count^#^	34.26 ± 29.11	27.05 ± 33.57	42.59 ± 33.12	NS	NS	**0.011**
CD8^high^CD57^+^CD27^−^						
%^4^	22.67 ± 13.33	25.52 ± 17.42	27.40 ± 19.55	NS	NS	NS
A. count^#^	150.6 ± 119.7	143.4 ± 157.7	155.2 ± 136.8	NS	NS	NS
CD8^high^CD57^−^CD27^+^						
%^4^	59.13 ± 17.54	46.51 ± 22.00	47.16 ± 22.29	**0.026**	NS	NS
A. count^#^	387.3 ± 211.4	227.9 ± 186.6	224.8 ± 129.3	**0.0003**	**0.005**	NS
CD8^high^CD57^−^CD27^−^						
%^4^	13.10 ± 8.112	22.60 ± 15.37	17.18 ± 11.20	**0.024**	NS	NS
A. count^#^	85.79 ± 73.99	119.1 ± 126.7	99.46 ± 85.96	NS	NS	NS
CD8^+^FoxP3						
%^5^	10.59 ± 15.16	5.881 ± 6.673	6.498 ± 7.939	NS	NS	NS
A. count^#^	91.42 ± 126.1	51.19 ± 86.72	50.49 ± 60.37	NS	NS	NS

^1^I group: healthy controls; ^2^II group: pSS patients without anti-SSA/SSB; ^3^III group: pSS patients with anti-SSA and/or anti-SSB; %^4^: proportion of all CD8^high^ lymphocytes; %^5^: proportion of all CD8^+^ lymphocytes. A. count^#^: absolute count of cells in 1 *μ*L of blood. ^*^Mann-Whitney test. NS: not significant.
